# Preliminary Investigation of the Diagnosis of Neonatal Congenital Small Bowel Atresia by Ultrasound

**DOI:** 10.1155/2019/7097159

**Published:** 2019-09-29

**Authors:** Ju Hao, Yao Zhang, Li Tianyu, Shi Bo, Feng Shu, Shi Feng, Ji Chao, Huang Ying

**Affiliations:** ^1^Department of Ultrasound, Shengjing Hospital of China Medical University, Shenyang 110004, China; ^2^Department of Pediatric Surgery, Shengjing Hospital of China Medical University, Shenyang 110004, China; ^3^Department of Medical Record, Shengjing Hospital of China Medical University, Shenyang 110004, China; ^4^Department of Clinical Epidemiology, Library, Shengjing Hospital of China Medical University, Shenyang 110004, China

## Abstract

**Purpose:**

To assess the diagnostic value of ultrasonography (US) for congenital small bowel atresia (SBA) in neonates and their sonographic characteristics.

**Methods:**

A retrospective analysis was performed of 20 neonates who were confirmed with SBA by operation from March 2014 to January 2019. All the neonates have been scanned by US before surgery, and no one underwent barium enema or upper gastrointestinal imaging prior to US. Preoperation ultrasound characteristics about intestinal morphology and intestinal contents were collected, further to summarize the typical ultrasonic features of SBA.

**Results:**

Five cases were duodenal atresia, and 15 cases were jejuno-ileal atresia. Distended proximal intestines, liquid with tiny points in it, can be found in 20 neonates. The small intestine without any gas can be found in 20 neonates. Microcolon, no gas and other contents in it, can be found in 16 cases.

**Conclusions:**

The typical ultrasonic features of SBA include dilation in proximal intestines, small intestines, and microcolon. US is a promising modality in the clinical diagnosis of SBA.

## 1. Introduction

Small bowel atresia (SBA) is one of the common causes of neonatal intestinal obstruction, with an incidence ranging from 1.3 to 2.8 out of 10,000 live births, including congenital duodenum atresia, jejunum atresia, and ileum atresia [1]. Plain abdominal radiographs can find intestinal obstruction, but they are not used for the diagnosis of SBA. Upper gastrointestinal and barium enema is helpful in the diagnosis of SBA [2], but it requires radiation and is an invasive examination. Although there can be interference by intestinal gas and stool mass, ultrasonography (US) is commonly used for the diagnosis and monitoring of inflammatory bowel diseases [3–7]. Being a safe, convenient, and inexpensive imaging method, US can be used to diagnose children's diseases of intussusception [8], malrotation of the intestine [9, 10], and intestinal polyp [11]. US is also suitable for neonates because of their thin abdominal wall, especially for neonates with SBA as there is little gas in the intestines. However, there are few ultrasound studies focused on SBA. In this study, we evaluated the data of neonates with SBA confirmed by surgery and summarized their ultrasonic image characteristics through preoperative examination, to provide a basis for the ultrasonic diagnosis of SBA.

## 2. Materials and Methods

### 2.1. Patients

A retrospective analysis was performed of 20 neonates who were admitted to the Pediatric Surgery Department of our hospital from March 2014 to January 2019, confirmed with duodenal atresia or jejuno-ileal atresia by operation. No sedatives were used during examination. No one underwent barium enema or upper gastrointestinal imaging prior to US.

This study was approved by the institutional review board of the hospital, and the requirement of informed consent was waived because of its retrospective nature.

### 2.2. Methods

Ultrasounds were performed using Philips iU22 Ultrasound System (Philips Healthcare, Bothell, WA, USA) or Toshiba Aplio400 (Toshiba Medical Systems Corporation, Tochigi, Japan) with 5 MHz–8 MHz convex array probe, 3 MHz–9 MHz linear array probe, and 5 MHz–12 MHz linear array probe.

Clinical data collected included general information (prenatal ultrasound diagnosis, age, sex, and term or preterm), symptoms (vomiting and passing meconium), signs (abdominal distention), and ultrasonic image characteristics (the morphology and contents of the intestines and the morphology and contents of the colon). Small bowel with a width greater than 17 mm was seen as intestinal dilation. The general information and sonographic characteristics of congenital SBA (including dilation in proximal intestines, small intestines without any gas, and microcolon) in neonates were assessed.

## 3. Results

The clinical data of 20 neonates with SBA are detailed in Table 1. Twenty neonates were scanned including 15 boys and 5 girls, born from 1 hour to 7 days: preterm 4 cases and term 16 cases. Five cases (25%) presented with vomiting. Twelve cases (60%) were fasting after they were born. Seven cases (35%) presented with distention, and 13 cases (65%) did not pass the meconium. Fourteen cases (70%) were suspected intestinal obstruction by prenatal diagnosis because of their obviously dilated intestines (small bowel with a width greater than 17 mm after 32 weeks of gestation).

**Figure 1:**
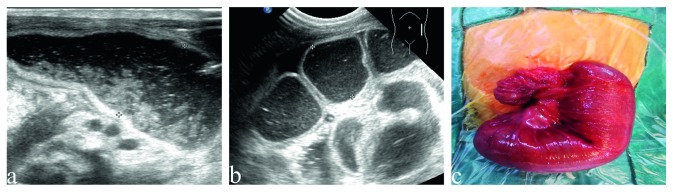
The dilated proximal intestines in neonates with small bowel atresia. (a) The dilated proximal intestines in neonates with duodenal atresia, liquid with tiny points in it. (b) The dilated proximal intestines in neonates with jejuno-ileal atresia, liquid with tiny points in it. (c) The dilated proximal intestines in operation.

**Figure 2:**
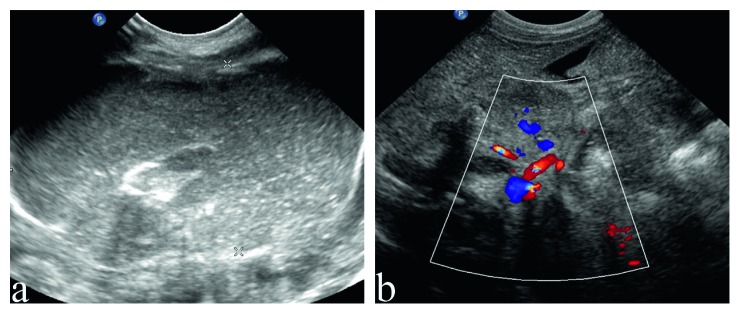
Jejunum atresia combined with rotation and meconium peritonitis. (a) Meconium peritonitis: a cystic mass with many little tiny points in abdominal. (b) Bowel rotation: the mesenteric blood vessels are entwined.

**Figure 3:**
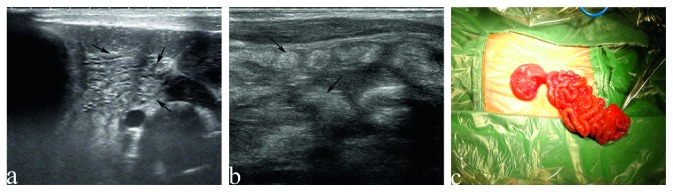
The small intestines in neonates with small bowel atresia. (a) The small intestines (black arrow) in neonates with duodenal atresia, with a little high echo in the center, no gas, are as small as an earthworm. (b) The small intestines (black arrow) in neonates with jejuno-ileal atresia, with a little high echo in the center, no gas, are as small as an earthworm. (c) The small intestines in operation.

**Figure 4:**
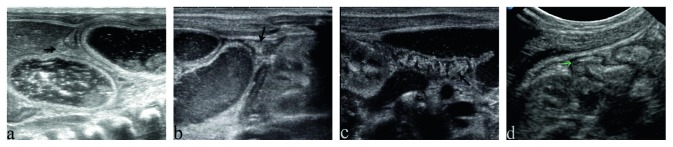
The microcolon in neonates with small bowel atresia. (a) The horizontal axis of ascending microcolon (black arrow) in neonates with duodenal atresia, very small and no gas in it. (b) The horizontal axis of descending microcolon (black arrow) in neonates with jejuno-ileal atresia, very small and no gas in it. (c) The vertical axis of microcolon (black arrow) in neonates, similar to appendix, nothing in it. (d) The empty ileocecal junction seems like a mushroom (green arrow).

The sonographic findings and postoperative findings are detailed in Table 2. Distended proximal intestines were found in all neonates during routine ultrasound examination. The width of these intestines ranges from 19 mm to 38 mm, full of liquid with tiny points in it (Figure 1). The terminal of distended intestines could be found through dilated bowel in 13 neonates. Six patients had meconium peritonitis, one of which combined with intestinal torsion (Figure 2). Cystic mass with many little tiny points was found in their abdominal; however, the terminal of distended intestines could not be identified. The small intestines, the distal intestines without any gas, were found in all neonates. They were about 5 mm in diameter, squiggly, as small as an earthworm (Figure 3). Only a small amount of fetal stool was present in these intestines, which appears to be hyperechoic on ultrasound ([Supplementary-material supplementary-material-1]). Microcolon was found in 16 cases (80%). With the width from 3 mm to 6 mm, these colons were similar to appendix and no gas was found in them. The empty ileocecal junction had the appearance of a mushroom sometimes (Figure 4).

Surgical treatment relieved the intestinal obstruction by anastomosing the proximal and distal intestines. Postoperatively, all the neonates survived after operation. Five neonates (25%) were confirmed with the diagnosis of duodenal atresia, and 15 neonates (75%) were confirmed with the diagnosis of jejuno-ileal atresia. Two neonates were confirmed as multiple atresia, which were not identified prior the operation. Six neonates (30%) had meconium peritonitis, one of them combined with intestinal torsion.

## 4. Discussion

### 4.1. Clinical Data Analysis

For SBA neonates, vomiting is a clinical characteristic as there is complete intestinal obstruction [12]. In this study, all the neonates born from 0 to 7 days were taken to the hospital. Fourteen cases (70%) were suspected by prenatal diagnosis, as a result, many of them were fed nothing and only 5 neonates performed vomiting. It is possible that prenatal diagnosis by ultrasound can reduce the postnatal vomiting rate by timely fasting. Abdominal distension, which is more obvious with lower obstruction, and complete abdominal distension are not possible with duodenal atresia. Failing to pass the meconium cannot be used to diagnose SBA, because some children are examined immediately after birth. Some children with SBA can also have fetal excretion, either meconium or necrotic tissue [2].

### 4.2. Analysis of Ultrasound Characteristics

In this study, the subsequent operation confirmed that 5 neonates were duodenal atresia and 15 neonates were jejuno-ileal atresia. There are many ultrasound images about duodenal atresia and jejuno-ileal atresia in common. Distended proximal intestines were easy to detect, which can also be found by prenatal diagnosis. In our study, 20 infants had distended proximal intestines with a width of 19 mm–38 mm. There was little gas in distended proximal intestines as there was full of swallowed amniotic fluid or breast milk. In addition, the lower the obstruction, the greater the number of distended loops of intestines will be observed. Thirteen patients were able to find the terminal of distended intestines, the location of the intestinal atresia (exactly the patient's first atresia), after careful examination through dilated intestines. The maximum dilatation of the proximal intestines usually occurs at the site of the obstruction. In our study, we could not find the terminal of distended intestines in 7 patients, because of the presence of meconium peritonitis and intestinal volvulus. Meconium peritonitis, a sterile chemical inflammation of the peritoneum from intestinal perforation in utero, usually presents as a meconium pseudocyst by prenatal ultrasound images [13]. In our study, meconium peritonitis presented as a cystic mass with many little tiny points consistent with literature reports. It is difficult to distinguish jejunum atresia and ileum atresia in some neonates by ultrasound examinations, because exact boundaries are difficult to discern between the jejunum and ileum on ultrasonic images.

Because of complete obstruction, little contents passed through the distal bowel. The small intestine, without any gas, is another characteristic of SBA. High echo can be seen in their center because there is a small amount of fetal stool. The amount of the small intestine that is affected is determined by the location of the atresia. It is most easy to find the small intestine in duodenum atresia patient, while it is difficult to find the small intestine in the ileum atresia patient.

Directly related to the fact that little meconium passes the obstruction area in the distal fetal intestine, the unused colon, is therefore not distended, no gas mostly, like an appendix. Microcolon is an important diagnostic basis for barium enema, and it also can be an important ultrasound manifestation as it can be found in 80% cases of SBA neonates. Microcolon could not be found in all neonates, possibly because the event leading to atresia occurred extremely late in gestation. Thus, the colon appeared of normal caliber. The mushroom sign, as a sonographic finding of ileocecal junction sometimes, signified the presence of the small intestine and microcolon at the same time.

When SBA was suspected, the distended intestines are easy to be found on sonographic examination. However, it is not a reliable indication independently, as it may be annular pancreas, intestinal stenosis, and congenital megacolon. US of the distal small intestines may be more helpful, especially if there is no gas present. Because the distal intestinal tube can contain more or less material and gas in most annular pancreas or intestinal stenosis patient, the megacolon neonate does not have the small intestine. However, the presence of distal intestinal gas in a neonate does not exclude the diagnosis of duodenal atresia, because air may bypass through anomalous ducts [14].

US is highly operator-dependent. Because of lack of awareness and experience, the small intestines and microcolon tend to be undetectable and the dilated intestine can easily be mistaken for colon. Barium enema or upper gastrointestinal imaging is usually used to find the terminal of distended intestines or microcolon, but it would make it difficult for US examination. It is better to take ultrasound examination before barium enema and upper gastrointestinal imaging.

### 4.3. Limitations of the Study

Our study had some limitations. The sample size was limited. This is a descriptive study as it is a case series and retrospective in nature. The number of neonates in whom SBA failed to be detected by ultrasonic examination (false negatives) was unknown. SBA cases and control groups were needed to calibrate our criteria for diagnosing intestinal atresia. Further research is needed, and more experience needs to be accumulated in order to better understand SBA.

## 5. Conclusions

The typical ultrasonic features of SBA include dilation in proximal intestines, small intestines, and microcolon. US is a promising modality in the clinical diagnosis of SBA.

## Figures and Tables

**Table 1:** The clinical data on 20 cases with small bowel atresia.

Case	Sex	Age	Term or preterm	Vomiting^*∗*^	Pass meconium^*∗∗*^	Distention^*∗∗∗*^	Fasting^*∗∗∗∗*^	Bowel dilation (prenatal ultrasound)^*∗∗∗∗∗*^
1	m	2 d	Term	+	–	–	–	–
2	m	1 h	Preterm	–	–	–	+	+
3	m	6 h	Term	–	+	–	–	+
4	m	1 d	Term	+	+	–	–	+
5	f	6 h	Term	–	+	+	+	+
6	m	23 h	Preterm	–	–	–	+	+
7	m	1 d	Term	–	–	–	+	+
8	m	19 h	Term	–	–	+	+	+
9	m	17 h	Term	–	–	–	+	+
10	m	5 d	Term	+	–	+	–	–
11	m	2 d	Term	–	+	–	–	–
12	f	9 h	Preterm	–	–	–	+	+
13	m	21 h	Preterm	–	–	–	+	+
14	f	2 h	Term	–	+	–	+	+
15	m	7 d	Term	+	+	–	–	–
16	f	1 d	Term	–	–	+	+	+
17	m	19 h	Term	–	–	+	–	–
18	m	23 h	Term	–	–	–	+	+
19	f	1 d	Term	+	–	+	–	–
20	m	8 h	Term	–	+	+	+	+

m: male; f: female; h: hour; d: day. ^*∗*^+: vomiting; –: no vomiting. ^*∗∗*^+: pass meconium; –: fail to pass meconium. ^*∗∗∗*^+: distention; –: no distention. ^*∗∗∗∗*^+: fasting; –: feeded with breast milk. ^*∗∗∗∗∗*^+: prenatal diagnosis of intestinal obstruction; –: no prenatal diagnosis of intestinal obstruction.

**Table 2:** The sonographic findings and postoperative findings in 20 cases with SBA.

Case	Sonographic findings						
	Intestinal dilation^*∗*^	Distended intestines' terminal^*∗∗*^	Small intestines^*∗∗∗*^	Microcolon^*∗∗∗∗*^	Colon (mm)	Cystic mass^*∗∗∗∗∗*^	Surgical findings
1	+	+	+	+	0.5	–	Ileum atresia
2	+	–	+	–	–	+	Ileum and jejunum atresia
3	+	+	+	+	0.5	–	Ileum atresia
4	+	+	+	+	0.5	–	Duodenal atresia
5	+	–	+	+	0.4	+	Jejunum atresia
6	+	+	+	+	0.5	–	Duodenal atresia
7	+	+	+	–	–	–	Duodenal atresia
8	+	–	+	+	0.5	+	Ileum atresia
9	+	–	+	+	0.4	+	Ileum atresia
10	+	–	+	+	0.5	–	Ileum atresia
11	+	–	+	+	0.4	+	Ileum atresia
12	+	+	+	–	–	–	Jejunum (multiple) atresia
13	+	+	+	+	0.5	–	Duodenal atresia
14	+	+	+	+	0.4	–	Jejunum atresia
15	+	+	+	+	0.6	–	Duodenal atresia
16	+	+	+	+	0.4	–	Jejunum atresia
17	+	+	+	+	0.5	–	Jejunum atresia
18	+	+	+	–	–	–	Jejunum atresia
19	+	+	+	+	0.4	–	Ileum atresia
20	+	–	+	+	0.3	+	Ileum atresia

^*∗*^+: some intestines diameter greater than 17 mm; –: no intestine diameter greater than 17 mm. ^*∗∗*^+: distended intestines' terminal can be found; –: distended intestines' terminal cannot be found. ^*∗∗∗*^+: small distal intestines can be found; –: small distal intestines cannot be found. ^*∗∗∗∗∗*^+: microcolon can be found; –: microcolon cannot be found. ^*∗∗∗∗∗*^+: cystic mass can be found; –: cystic mass cannot be found.

## Data Availability

The data used to support the findings of this study are available from the corresponding author upon request.

## Supplementary Materials

Supplementary Materials Video 1: during the operation, a little white fetal stool was squeezed out of the small intestine.
